# Baicalin-Induced Autophagy Preserved LPS-Stimulated Intestinal Cells from Inflammation and Alterations of Paracellular Permeability

**DOI:** 10.3390/ijms22052315

**Published:** 2021-02-26

**Authors:** Valentina Rizzo, Nadia Ferlazzo, Monica Currò, Gaetano Isola, Marco Matarese, Maria Paola Bertuccio, Daniela Caccamo, Giovanni Matarese, Riccardo Ientile

**Affiliations:** Department of Biomedical and Dental Sciences and Morphofunctional Imaging, University of Messina, Polyclinic Hospital University, 98158 Messina, Italy; rizzo_valentina@hotmail.com (V.R.); nferlazzo@unime.it (N.F.); gaetano.isola@unict.it (G.I.); matamarco94@gmail.com (M.M.); mp.bertuccio@gmail.com (M.P.B.); dcaccamo@unime.it (D.C.); gmatarese@unime.it (G.M.); ientile@unime.it (R.I.)

**Keywords:** autophagy, baicalin, claudin 1, inflammatory bowel diseases, Nuclear Factor-κB, paracellular permeability

## Abstract

Several studies have demonstrated a relevant role of intestinal epithelial cells in the immune response and in chronic inflammatory conditions, including ulcers, colitis, and Crohn’s disease. Baicalin (BA), extracted from the root of Scutellaria baicalensis, has various beneficial healthy effects, including anti-inflammatory activity. However, few studies have evaluated BA effects on autophagic signaling in epithelial cell response to inflammatory stimuli. To explore possible beneficial effects of BA, HT-29 cells were exposed to lipopolysaccharide (LPS), in presence or absence of BA, for 4 h. We evaluated mRNA levels of autophagy-related genes and cytokines, triggering inflammatory response. Furthermore, the expression of claudin 1, involved in the regulation of paracellular permeability was analyzed. BA treatment repressed LPS-induced expression of TNF-α and IL-1β. The down-regulation of autophagy-related genes induced by LPS was counteracted by cell pretreatment with BA. Under these conditions, BA reduced the NF-κB activation caused by LPS. Also, BA restored mRNA and protein levels of claudin 1, which were reduced by LPS. In conclusion, in intestinal epithelial cells BA regulates the NF-κB activation and modulates both autophagic and inflammatory processes, leading to an improvement of paracellular permeability. These results suggest that the anti-inflammatory effects of BA can be associated to the regulation of autophagic flux.

## 1. Introduction

Epithelial cells of intestinal mucosa are essential for defense against different stimuli, such as pathogens, damaged cells, and food components, which can trigger inflammatory responses [1]. The dysregulation of immune functions of intestinal epithelial cells leads to the development of chronic inflammatory conditions, such as ulcers, colitis, Crohn’s disease, and mucositis [1].

Autophagy is a physiological catabolic process of cytoplasmic materials, including aggregated proteins, damaged organelles or pathogens, inside the lysosome, which contributes to the maintenance of cellular homeostasis [2]. In addition, it has been demonstrated that dysregulation of autophagy is involved in chronic intestinal inflammation [3]. Moreover, signaling molecules of this mechanism may be useful for monitoring alterations of paracellular permeability, promoting intestinal tissue damage [4,5].

Recent evidence from investigations in both mice and humans shows that inflammation in intestinal diseases can be alleviated by restoring impaired autophagy [6]. In this regard, many efforts have been focused on the identification of natural molecules acting as autophagy modulators in order to prevent the damage of intestinal mucosa associated with the inflammatory cascade [7].

Extract of *Scutellaria baicalensis Georgi* (SB), a perennial herb widespread especially in the regions of East Asia, is largely used by Traditional Chinese Medicine for its beneficial action on health, since it displays anti-inflammatory, anti-cancer, antibacterial and antiviral, antioxidant, and neuroprotective effects [8]. These properties may be ascribed to several flavonoids and phytosterols isolated from SB, such as baicalin (BA), baicalein, β-sitosterol, oroxylin, wogonin, and wogonoside [9,10].

Recently, it has been demonstrated that BA exerts anti-inflammatory properties in animal model of ulcerative colitis (UC), thus suggesting that BA may be useful to relieve the symptoms of human intestinal inflammatory conditions [11].

However, the possible effects of BA as modulatory of autophagy in intestinal epithelial cells have not been investigated yet. Therefore, the present study was aimed to determine whether the anti-inflammatory effects of BA are associated with changes in autophagy pathway in HT-29, a human colonic epithelial cell line, stimulated by LPS as in vitro model of inflammation [12,13,14]. In order to evaluate the potential benefits of BA, we investigated changes in autophagy biomarkers by also exploring the activation status of NF-κB and the expression of claudin 1, a tight-junction (TJ) protein involved in cell-to-cell adhesion.

## 2. Results

### 2.1. Cell Viability 

**Figure 1:**
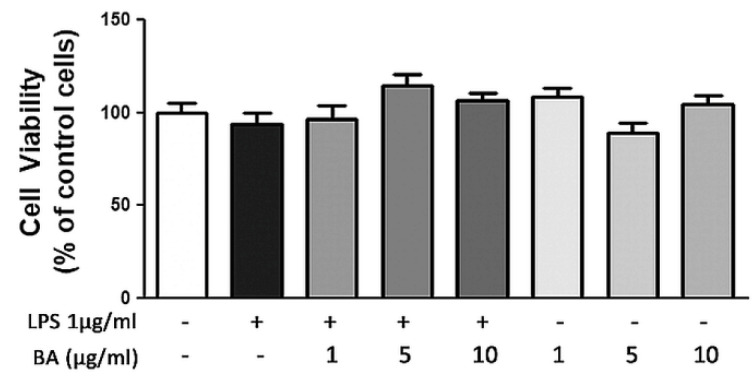
Effects of BA on HT-29 cell viability in presence or absence of LPS. BA was added to the culture medium 30 min before LPS treatment for 4 h. Then, cell viability was assessed by MTT assay.

In order to study BA toxicity on cell cultures, preliminary experiments were carried out using MTT test. As shown by MTT results, the exposure of HT-29 cells to different BA concentrations (in a range 1–10 µg/mL), in presence or absence of LPS at 1 µg/mL for 4 h, did not show any significant variation of cell viability (Figure 1). 

### 2.2. Gene Expression and Release of Cytokines

**Figure 2:**
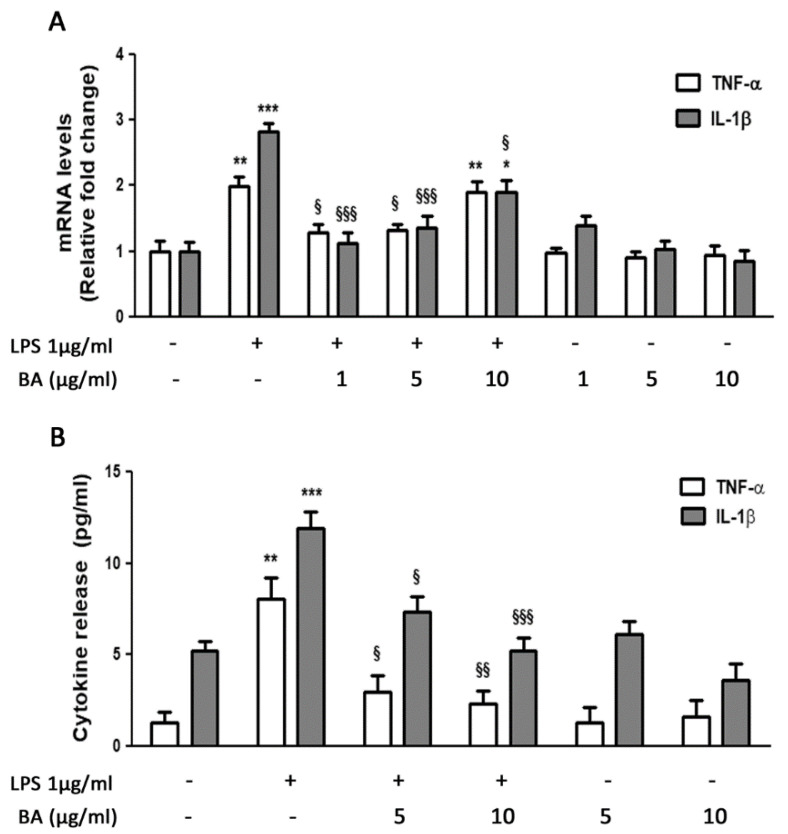
Effects of BA on cytokine gene transcription and release in HT-29 cells stimulated with LPS. (A) HT-29 cells were treated with BA that was added to culture medium 30 min before exposure to LPS for 4 h. The mRNA transcript levels were analyzed by Real-Time PCR. (B) Cytokine levels in culture medium were measured by ELISA. * *p* < 0.05, ** *p* < 0.01, *** *p* < 0.001, significant differences vs. Ctr; ^§^ *p* < 0.05, ^§§^ *p* < 0.01, ^§§§^ *p* < 0.001, significant differences vs. LPS-treated cells.

Real-Time PCR analysis showed a significant increase in TNF-α and IL-1β transcript levels following stimulation with LPS (1 μg/mL) compared with control cells. In particular, we observed a twofold and a threefold increase for TNF-α and IL-1β, respectively. The pretreatment with BA was effective in counteracting the inflammatory response induced by LPS. In samples treated with BA, we found a significant reduction of both pro-inflammatory cytokines, TNF-α and IL-1β, compared with samples treated with LPS alone. In particular, the pretreatment with BA at concentrations of 1 and 5 μg/mL reduced the IL-1β mRNA levels by approximately 35% and TNF-α mRNA levels by about 50%, when compared with LPS-treated cells. Surprisingly, the highest concentration of BA (10 μg/mL) was able to significantly reduce only IL-1β transcript levels (35%, *p* < 0.05), while it did not affect TNF-α levels, in comparison to LPS-treated cells. Treatment of cells with BA alone did not produce any effect on the expression values of both cytokines that were similar to those reported in control cells (Figure 2A). The effects of BA on the expression of pro-inflammatory cytokines were confirmed by the analysis of cytokine release in the culture medium. As shown in Figure 2B, the pretreatment with BA at different concentrations significantly reduced the LPS-induced release of both TNF-α and IL-1β, also when used at 1 μg/mL (data not shown). The slight discrepancy between mRNA and protein release may be due to several processes occurring between transcription and translation. 

### 2.3. Gene Expression of Autophagy-Related Genes

**Figure 3:**
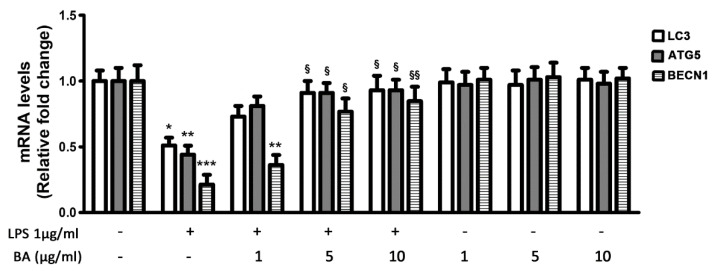
Effects of BA on LC3, ATG5 and BECN1 gene expression in HT-29 cells stimulated with LPS. HT-29 cells were treated with BA that was added to culture medium 30 min before exposure to LPS for 4 h. The mRNA transcript levels were analyzed by Real-Time PCR. * *p* < 0.05, ** *p* < 0.01, *** *p* < 0.001, significant differences vs. Ctr; ^§^ *p* < 0.05, ^§§^ *p* < 0.01, significant differences vs. LPS-treated cells.

The expression of LC3, ATG5 and BECN1 genes was significantly down-regulated by LPS treatment (Figure 3), and this effect was counteracted by cell pretreatment with BA. The BA concentration of 1 μg/mL was less effective than the other tested concentrations, since it increased the mRNA levels of LC3 and ATG5 in comparison with LPS-treated cells, but it only slightly affected the expression of BECN1 that remained significantly reduced (−60%) in comparison with untreated cells. Instead, the BA concentrations of 5 and 10 μg/mL were able to restore the expression of either LC3, ATG5 or BECN1 genes at levels similar to those of control cells (Figure 3).

### 2.4. Inhibition of LPS-Induced NF-κB Activation 

**Figure 4:**
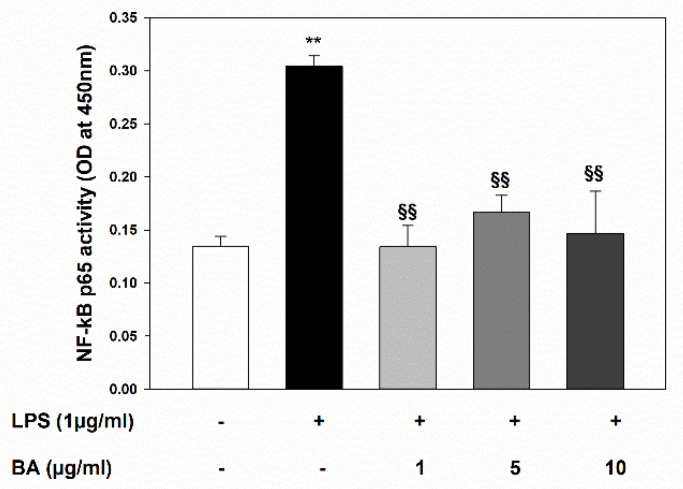
Inhibitory effect of BA on LPS-induced NF-κB p65 activation in HT-29 cells. Cell cultures were treated with BA that was added to culture medium 30 min before incubation with LPS for 4 h. NF-κB p65 activation was determined by ELISA performed on nuclear extracts. ** *p* < 0.01 significant differences vs. Ctr; ^§§^ *p* < 0.01 significant differences vs. LPS treated cells.

In order to characterize the underlying mechanisms involved in BA anti-inflammatory effects on intestinal epithelial cells, we investigated the activation status of NF-κB in HT-29 cell response to LPS-induced injury. As shown in Figure 4, the LPS treatment caused an intense activation of NF-κB, which was suppressed by pre-incubation with all tested BA concentrations.

### 2.5. Expression of TJ Protein Claudin 1 

**Figure 5:**
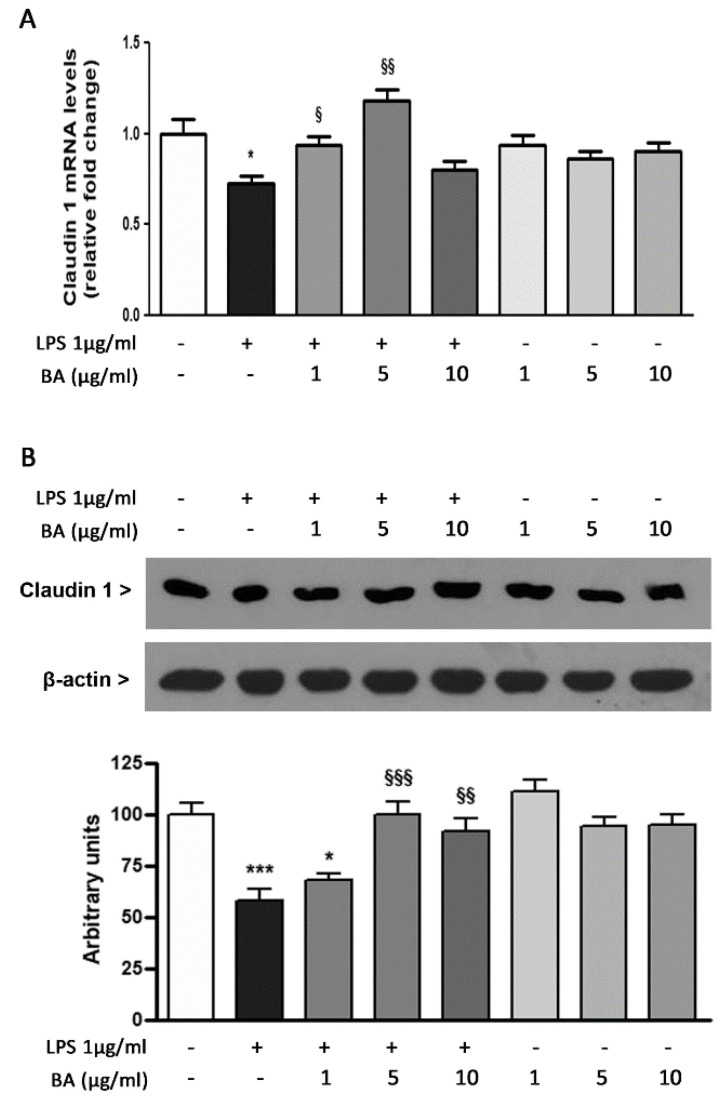
Effects of BA on claudin 1 gene and protein expression in HT-29 cells treated with LPS. (**A**) HT-29 cells were treated with BA that was added to culture medium 30 min before exposure to LPS for 4h. The mRNA transcript levels were analyzed by Real-Time PCR. (**B**) The expression levels of claudin 1 protein were evaluated by Western blotting. The densitometric analysis (bottom) of claudin 1 band intensities was performed after protein normalization against β-actin levels. * *p* < 0.05, *** *p* < 0.001, significant differences vs. Ctr; ^§^ *p* < 0.05, ^§§^ *p* < 0.01, ^§§§^ *p* < 0.001, significant differences *vs*. LPS.

The possible effect of BA on cell–cell interactions has been evaluated by analyzing the expression of claudin 1, involved in the control of paracellular permeability through intercellular spaces. As shown in Figure 5A, the transcription level of claudin 1 gene was down-regulated by about 30% following stimulation with LPS. This effect was reduced by the pretreatment of HT-29 cells with BA. Indeed, the incubation with BA in presence of LPS restored the expression levels of claudin 1 at values similar to those observed in control cells. In this case, the highest concentration of BA (10 µg/mL) was the least effective. The analysis of claudin 1 expression in cells incubated with BA alone showed no significant differences with respect to control cells. These results were confirmed by Western blot analysis (Figure 5B).

## 3. Discussion

The possible use of BA, either administered alone or in combination with other agents, has recently been suggested for the treatment of several inflammatory diseases [15,16,17]. In an in vivo model of UC, BA administration by intragastric injection reduced symptoms of colon inflammation, such as disease activity index and histological scores. Under these conditions, BA also modulated the expression of both pro-inflammatory and anti-inflammatory cytokines, by blocking TLR4/NF-κB signal transduction pathway [11]. Zhu and coworkers [18] demonstrated the effectiveness of BA against dextran sulfate sodium (DSS)-induced colitis throughout the modulation of macrophage polarization to the M2-activated phenotype. These events are primarily involved in tuning inflammatory responses and promoting tissue remodeling and repair. According to previous data, the results of present study demonstrated a powerful anti-inflammatory effect of BA against LPS-induced stimulation of HT-29, a colon-derived cell line. Indeed, a significant reduction in mRNA transcript levels and release of cytokines IL-1β and TNF-α was evidenced in HT-29 cells pretreated with BA in comparison to LPS-treated cells. 

It has been clearly demonstrated that the aberrant production of inflammatory cytokines, such as TNF-α, IL-1β and IL-6, often observed in the gut of patients with inflammatory bowel disease (IBD), is a consequence of deregulated NF-κB signaling cascade [19]. A recent report investigating the underlying mechanisms of anti-inflammatory properties of BA showed that BA suppressed the IKB/NF-κB signaling pathway in RAW264.7 macrophages stimulated with LPS. The same mechanism has been observed in trinitrobenzene sulfonic acid (TNBS)-induced UC rat model [20]. It has also been reported that BA reduced reactive oxygen species (ROS) production and suppressed apoptosis through inhibition of NF-κB and NLRP3 signaling pathways in mononuclear phagocytes of piglets infected by a bacterium causing membrane inflammation [21]. Furthermore, BA attenuated inflammation and LPS-induced apoptosis in cattle mammary epithelial cells through inhibition of NF-κB activation and HSP72 up-regulation [22]. Here we report that also in intestinal epithelial cells LPS-induced NF-κB activation was suppressed by BA addition, confirming that BA anti-inflammatory effects involve the inhibition of NF-κB. 

In recent years, it has been reported an essential role of autophagy in innate and adaptive immune response, such as antigen presentation, cytokine secretion and antimicrobial peptide production [23]. The role of autophagy in intestinal inflammation and restoring intestinal homeostasis has also been demonstrated [6]. In addition, a crosstalk between NF-κB and autophagy has been reported both in in vitro experimental models and in colon tissues of patients with UC [24]. In particular, LPS treatment induced NF-κB activation and repressed the autophagic flux in Caco-2 and HT-29 cells, while the knockdown of NF-κB p65 subunit restored autophagy protein levels. In parallel, the inhibition of ATG5 exacerbated LPS-induced inflammation, while the administration of autophagy stimulators reduced the severity of experimental colitis in mice [24].

Using an in vitro model, we demonstrated that BA treatment was able to restore the expression levels of some autophagy-related genes, and this effect was associated with NF-κB inhibition. According to previous results [24], our data indicate that in intestinal epithelial cells BA treatment is associated with reduction of NF-κB activation, which, in turn, promotes an improvement of autophagic flux. However, further in vivo experimental investigations will be useful to confirm that BA extract is effective in preserving intestinal cells from inflammation by improving autophagy.

The inflammatory cascade can produce a series of alterations, such as perturbation of TJ, intestinal epithelial cell injury and crypt abscesses, leading to an increase in intestinal permeability [25,26]. In this context, autophagy pathway defects in intestinal epithelial cells increase susceptibility to inflammation, leading to cell death and intestinal epithelial barrier breakdown [27,28]. 

Claudin 1 is a trans-membrane protein of TJ [29], that together with occludin and cytosolic proteins, such as zonulin 1, contributes to the maintenance of paracellular permeability. Claudins with desmosomes, adherens junctions and other TJ, hold together the cells that compose the intestinal epithelial barrier [30]. Numerous studies have reported a reduced expression of claudin 1 in patients with IBD [31]. In our study, the inflammatory response induced by exposure of HT-29 cells to LPS was accompanied by a reduction in claudin 1 expression. Interestingly, this effect was rescued by pretreatment with BA, suggesting that the anti-inflammatory properties of BA may be associated with a beneficial action on intestinal barrier integrity, although further characterization of underlying molecular pathways is necessary.

BA is used as an anti-inflammatory drug in traditional Chinese medicine, but nowadays SB extracts are available in different commercial formulations also in other countries. Despite the fact that BA is considered clinically safe, previous data have demonstrated the toxicity of BA administration in rodents and dogs [32,33]. Recently, Cai and collaborators [34] demonstrated that high doses of baicalin induced renal fibrosis in Sprague-Dawley rats.

In light of these observations, the side effects and toxicity of BA should be better defined. In addition, given the widely reported properties of bioactive compounds to exert biphasic dose-responses depending on their concentration [35,36,37,38], it can be hypothesized that also BA possesses hormetic properties. In this regard, a recent study reported a dual effect of BA on angiogenic process in chick embryo chorioallantoic membrane model; in particular, BA at low doses stimulated the angiogenic process, while at high doses displayed inhibitory effects [39]. In line with this observation, in our experimental model, low doses of BA (1–5 µg/mL) showed protective effects against LPS-induced inflammatory response and intercellular TJ alteration, while a higher concentration (10 µg/mL) of BA was less effective.

Overall, our observations highlight the beneficial effects of BA in inflamed intestinal epithelial cells and the involvement of a crosstalk between autophagy and NF-κB pathway in the modulation of inflammatory cell response and intercellular interactions. These findings suggest that BA can be used as supplemental therapy for intestinal inflammatory conditions in order to reduce the inflammatory response induced by chronic stress of our intestine. However, further pre-clinical and clinical studies are needed in order to understand BA safety, bioavailability, bioefficacy, and possible drug–drug interactions in patients with intestinal inflammatory diseases.

## 4. Materials and Methods

### 4.1. Materials

The human colonic epithelial cell line, HT-29, was purchased from Sigma Aldrich (Milan, Italy). RPMI-1640, L-glutamine, HEPES, sodium pyruvate, glucose, 2-mercaptoethanol, penicillin/streptomycin mixture, dimethyl sulfoxide (DMSO), lipopolysaccharide (LPS), 3-(4,5-methylthiazol-2-yl)-2,5-diphenyl-tetrazoliumbromide (MTT), phosphate buffered saline solution (PBS), was from InvivoGen (San Diego, CA, USA). Fetal bovine serum (FBS), as well as TRIzol, High-capacity cDNA archive kit, SYBR Select Master Mix, the biotin 3′-end DNA labeling kit, LightShift Chemiluminescent Electrophoretic Mobility Shift assay (EMSA) kit, Biodyne Nylon membranes, and Super-Signal West Pico chemiluminescent substrate system were from Life Technologies (Milan, Italy). Specific primers for Real-Time PCR were from Eurofins Genomics (Ebersberg, Germany). ELISA kits for the quantitative detection of human TNF-α and IL-1β were from Cloud-Clone Corp. (Katy, TX, USA). BA was provided by Enfarma SRL (Misterbianco, Italy) [13]. 

BA (SB root dry extract titrated at 95% in BA) was dissolved in DMSO in a 10 mg/mL stock solution, stored at −20 °C, and diluted to different concentrations with culture medium right before experimental use.

### 4.2. Cell Culture and Ttreatment

HT-29 cells were maintained in RPMI 1640 supplemented with L-glutamine (2 mM), HEPES (10 mM), sodium pyruvate (1 mM), 2-mercaptoethanol (0.05 mM), 1% penicillin/streptomycin, and 10% FBS, at 37 °C in a 5% CO_2_/95% air humidified atmosphere. Medium was changed every 2 days, and split-performed when cells reached maximum density of about 80%. In our experimental conditions, HT-29 cells were seeded at a density of 3 × 10^5^ cells/mL into culture plates in RPMI complete medium plus 10% FBS and incubated at 37 °C with LPS (1 µg/mL) for 4 h, in presence or absence of BA (1-5-10 µg/mL), which was added to the culture medium 30 min prior to LPS treatment. In all experiments, equal volumes of PBS or DMSO were added to the medium of control cells. 

LPS concentration to be used throughout the experiments was chosen following preliminary dose-response and time-dependent assays. In particular, we tested the effects of 0.1-0.5-1 µg/mL LPS after 1-4-8-16-24 h of incubation on cytokine production. A concentration of 1 μg/mL LPS after 4 h resulted the most effective to evoke significant increases of cytokine mRNA levels.

After incubation, cells were used for further assays and media were collected in order to evaluate cytokine release.

### 4.3. Cell Viability Assay

The LPS and BA effects on cell viability were evaluated by a MTT quantitative colorimetric assay, as previously described [40]. After treatment, HT-29 plated at a density of 2 × 10^4^ cells/well in 96-well culture cells were incubated with fresh red-phenol free medium containing MTT (0.5 mg/mL) at 37 °C for 4 h. Then, insoluble formazan crystals were dissolved in 100 µL of a 0.04 N HCl/isopropanol solution for 1 h. The optical density in each well was evaluated by spectrophotometrical measurement at 570 nm using a microplate reader (Tecan Italia, ColognoMonzese, Italy). All experiments were performed in triplicate.

### 4.4. Real-Time PCR

Quantitative Real-Time PCR has been performed as previously reported [41]. Briefly, after RNA isolation with TRIzol reagent, RNA (2 µg) was reverse-transcribed with High-Capacity cDNA Archive kit according to the manufacturer’s instructions. Then, mRNA levels of TNF-α, IL-1β, LC3, ATG5, BECN1 and Claudin 1 were analyzed by Real-Time PCR using SYBR green-based gene expression analysis. β-actin was used as endogenous control. Quantitative PCR reactions were set up in triplicate in a 96-well plate, and carried out in a final volume of 10 µL, containing 1× SYBR Select PCR Master Mix, 0.1 µM specific primers, and 25 ng cDNA. The primer sequences used are reported in Table 1. qRT-PCR was performed in a 7900HT Fast Real-Time PCR System (Applied Biosystems, Foster City, CA, USA) with the following profile: one cycle at 95 °C for 10 min, followed by 40 cycles at 95 °C for 15 s and 60 °C for 1 min. A standard dissociation stage was added to assess primer annealing specificity. Data were collected and analyzed using SDS 2.3 and RQ manager 1.2 software (Applied Biosystems, Foster City, CA, USA) using the 2(^−ΔΔCt^) relative quantification method. Values are presented as fold change relative to untreated cells.

### 4.5. Evaluation of Cytokine Secretion by ELISA

In order to detect human IL-1β and TNF-α, an enzyme linked immunosorbent assay was performed in cell-free culture supernatants of HT-29 cells, using ELISA Kits according to the manufacturer’s guidelines. Before detection, supernatants recovered from treated and untreated cells were concentrated 10-fold by freeze-drying. All freeze-dried samples were reconstituted by the addition of distilled water. Briefly, 50 µL of standards or samples were incubated in 96-well plates at room temperature for 3 h with shaking. After washing 5 times with 400 µL of Wash buffer, 100 µL of the provided substrate solution were added to each well, and the plates were incubated in the dark for 10 min. The enzyme reaction was then stopped by pipetting 100 µL of stop solution into each well and the absorbance was determined at 450 nm using a microplate reader (Tecan, Italy). All experiments were performed in triplicate.

### 4.6. Colorimetric NF-κB Assay

The NF-κB assay was performed using TransAM Active Motif Kit according to THE manufacturer’s instructions (Active Motif, Waterloo, Belgium). The assay is based on enzyme-linked immunosorbent assay (ELISA) principle with colorimetric detection and relative quantification of activated NF-κB by spectrophotometry. The absorbance was read on a spectrophotometer at 450 nm.

### 4.7. Western Blot 

Protein expression has been analyzed as previously described [42]. Briefly, to obtain whole cell extracts, cells were lysed using ice-cold RIPA buffer supplemented with Protease Inhibitor Cocktail (SIGMA Aldrich, Milan, Italy) and cell debris were removed by centrifugation at 8000× *g* at 4 °C for 20 min. Protein concentrations were determined using a Bradford Assay and 30 μg of protein samples were separated by SDS-PAGE onto 10% gel, and transferred to nitrocellulose membranes. Then, the membranes were blocked with 5% non-fat dry milk in Tris-buffered saline containing 0.15% Tween 20 (TBS-T) for 1 h at room temperature. The membranes were probed with mouse monoclonal antibody against β-actin (diluted 1:3000 in TBS-T), and with rabbit monoclonal antibody against claudin 1 (diluted 1:500 in TBS-T), followed by incubation with HRP-conjugated anti-mouse (1:10,000 against anti-β-actin in TBS-T) and anti-rabbit (1:3000 against anti-claudin 1 in TBS-T) secondary antibodies. Immunoblots were developed with ECL detection system kit using Kodak film. The bands were scanned and quantified by densitometric analysis with ImageJ 1.45 software using β-actin for normalization. 

### 4.8. Statistical Analysis

Data obtained from three separate experiments were expressed as mean ± SEM, and analyzed by one-way analysis of variance (ANOVA), followed by the Bonferroni post hoc test using GraphPad Prism software version 5 (San Diego, CA, USA). P values lower than 0.05 were considered significant.

## Figures and Tables

**Table 1:** Primers used for Real-time PCR analysis of gene expression.

Target	Primer Sequence 5′>3′	
	Forward	Reverse
TNF-α	GTGAGGAGGACGAACATC	GAGCCAGAAGAGGTTGAG
IL-1β	GCTTATTACAGTGGCAATGA	TAGTGGTGGTCGGAGATT
LC3	CGGTGATAATAGAACGATACAAG	CTGAGATTGGTGTGGAGAC
ATG5	TGCCTGAACAGAATCATCCTT	CCAGCCCAGTTGCCTTAT
BECN1	ACAGTGAACAGTTACAGATGGA	CTCAGCCTGGACCTTCTC
Claudin 1	GTTGGGCTTCATTCTCGCCTT	CCTGGGCGGTCACGATGTTGTC
β-actin	TTGTTACAGGAAGTCCCTTGCC	ATGCTATCACCTCCCCTGTGTG

## Data Availability

The data presented in this study are available in the article.
